# 

**Published:** 1890-04-26

**Authors:** 


PRICE ONE PENNY.

No. 187, Vol. VIII.—April 26th, 1890.

[Registered at the General Post Office as a Newspaper for transmission in the United Kingdom and Abroad.]

WITH EXTRA NURSING SUPPLEMENT.
Per Annum, 6s. 6d.; post free.
Monthly Parts, 6d.; post free 8d.
Ditto per Annum, 8s. post free.

